# MUF-Net: A Novel Self-Attention Based Dual-Task Learning Approach for Automatic Left Ventricle Segmentation in Echocardiography †

**DOI:** 10.3390/s25092704

**Published:** 2025-04-24

**Authors:** Juan Lyu, Jinpeng Meng, Yu Zhang, Sai Ho Ling

**Affiliations:** 1 College of Artificial Intelligence, Tianjin University of Science and Technology, Tianjin 300457, China; lvjuan@tust.edu.cn; 2 College of Light Industry Science and Engineering, Tianjin University of Science and Technology, Tianjin 300457, China; 22062212@mail.tust.edu.cn; 3 School of Electrical and Data Engineering, Faculty of Engineering and Information Technology, University of Technology Sydney, Sydney, NSW 2007, Australia; steve.ling@uts.edu.au

**Keywords:** self-attention, left ventricular segmentation, optical flow, spatio-temporal feature

## Abstract

Left ventricular ejection fraction (LVEF) is a critical indicator for assessing cardiac function and diagnosing heart disease. LVEF can be derived by estimating the left ventricular volume from end-systolic and end-diastolic frames through echocardiography segmentation. However, current algorithms either focus primarily on single-frame segmentation, neglecting the temporal and spatial correlations between consecutive frames, or often fail to effectively address the inherent challenges posed by the low-contrast and fuzzy edges characteristic of echocardiography, thereby resulting in suboptimal segmentation outcomes. In this study, we propose a novel self-attention-based dual-task learning approach for automatic left ventricle segmentation. First, we introduce a multi-scale edge-attention U-Net to achieve supervised semantic segmentation of echocardiography. Second, an optical flow network is developed to capture the changes in the optical flow fields between frames in an unsupervised manner. These two tasks are then jointly trained using a temporal consistency mechanism to extract spatio-temporal features across frames. Experimental results demonstrate that our model outperforms existing segmentation methods. Our proposed method not only enhances the performance of semantic segmentation but also improves the consistency of segmentation between consecutive frames.

## 1. Introduction

Cardiovascular diseases are the leading cause of death worldwide, accounting for 32% of all deaths, with heart attacks and strokes contributing to 85% of this mbox deaths [1]. Consequently, monitoring heart health and diagnosing cardiovascular diseases is of paramount importance. LVEF is a crucial index for the evaluation of heart health, calculated from left ventricular volumes at end-systole (ES) and end-diastole (ED) [2]. Echocardiography is a widely used tool for evaluating cardiac function and structure at any clinical stage [3]. Typically, physicians identify the ES and ED frames from the patient’s echocardiogram video and subsequently calculate the LVEF using the formula LVEF = 1 − ESV/EDV [2]. However, this method is highly inefficient, as an echocardiography may contain dozens of frames or more, and examining each frame individually significantly reduces the physician’s work efficiency. In addition, the results obtained may be inaccurate due to the fuzzy edges and high noise levels on echocardiograms, which can prevent physicians from accurately delineating image boundaries and lead to mischaracterized images. Consequently, the development of an automated, robust, and highly accurate method for ES and ED frame identification is essential to enhance the efficiency and reliability of echocardiographic analysis.

In recent years, the rapid advancement of deep learning has led to its widespread application in various fields of medical imaging, with medical image segmentation emerging as one of the most important applications. Deep-learning segmentation algorithms for echocardiograms can be broadly categorized into two main types: single-frame segmentation and echocardiogram video segmentation. Single-frame segmentation methods focus exclusively on ES and ED frames without considering temporal information or inter-frame correlations. For instance, papers [4,5] employ a U-Net-based network to segment ES and ED frames. Li et al. proposed a multi-level, multi-scale dense pyramid and Deep Supervision Network (DPSN) for keyframe segmentation in multi-chamber views [6]. Yang et al. introduced the Efficient Pyramid Pooling (EASPP) module, which performs feature fusion through convolution with different dilation rates and global pooling [7]. These methods are used to extract features by enlarging the receptive field or to enhance the segmentation ability of the left ventricle by multi-scale feature fusion. However, convolution operations are limited in their ability to capture global image relationships, and pooling can lead to the loss of important information. In addition, enlarging the local receptive field may cause the segmentation results to be affected by speckle noise. Other methods [8,9] integrate a Convolutional Neural Network (CNN) with a transformer module, using image patches for segmentation. While transformer [10] can capture long-range dependencies in sequences, segmentation results using this approach are susceptible to noise. Subsequently, the vision transformer [11], tailored for computer vision tasks, was proposed. This method involves cutting the feature map into patches, treating each patch as a sequence to capture dependencies between sequences. Although positional encoding is incorporated in the embedding process, the arrangement of pixels within patches can still be affected when reconstructing the feature map, which affects the feature extraction. Some researchers have also introduced attention mechanisms, such as bridge attention [9] and attention refinement modules [12], to enhance feature fusion in segmentation. However, these methods do not specifically address the fuzzy edges characteristic of echocardiographic images. Moreover, single-frame segmentation methods typically ignore the temporal information and inter-frame correlations, making it challenging to accurately delineate the left ventricular region.

Recently, there has been a growing interest in echocardiogram video segmentation, which identifies ES and ED frames based on the volume obtained from the segmentation of all frames. To incorporate temporal information, some methods employ 3D structures to simultaneously extract semantic and temporal features. For example, Wei et al. proposed a co-learning network based on a 3D U-Net, trained at both the appearance and shape levels [13]. Chen et al. introduced a 3D U-Net method for echocardiogram video segmentation, which learns ED and ES segmentation while simultaneously tracking motion between frames [14]. However, 3D-based networks are not suitable for single-image cases, limiting their clinical applicability. Other approaches utilize a 2D plus time (2D + t) architecture to capture spatio-temporal information from video or image sequences. Li et al. proposed a multi-view echocardiography video segmentation network based on Long Short-Term Memory (LSTM), named MV-RAN [15]. Although MV-RAN can simulate temporal consistency, the LSTM structure is computationally intensive, and the performance at the end of the video is worse than at the beginning due to accumulated errors. Sirhani et al. proposed an EchoRCNN model based on Mask Region CNN (Mask R-CNN) [16]. Although LSTM and Mask R-CNN can capture the relationship between frames, LSTM will continuously accumulate errors, and Mask R-CNN needs to delineate the mask image of the first frame, which increases the cost of clinical application. Additionally, EchoRCNN has been validated on a relatively small dataset of only 750 videos. Painchaud et al. proposed a forced temporal consistency post-processing method for echocardiogram video segmentation [17], but its performance improvement is limited. Wu et al. introduced an Adaptive Spatio-temporal Semantic Calibration (ASSC) module, which leverages the spatio-temporal information between consecutive frames and overcomes the shortcomings of optical flow-based models that are sensitive to speckle noise [18]. However, the ASSC module employs a series of transformations and introduces several learnable transformation metrics for coordinate distortion calibration and channel feature weighting calibration, which increases model complexity and makes it more challenging to learn these metrics.

To address the aforementioned challenges, we propose a novel self-attention-based dual-task learning approach for left ventricular segmentation in echocardiography, termed MUF-Net. Firstly, to enhance the segmentation capability for the fuzzy edges of the left ventricle, we introduce a multi-scale edge-attention U-Net model (MSEA-U-Net). In its encoder stage, convolutional kernels with varying dilation rates and a self-attention mechanism are employed to perform multi-scale fusion of deep semantic features and capture long-range pixel-level dependencies within the feature map. Subsequently, in the decoder stage, the Sobel edge detection operator combined with a coordinate attention mechanism is utilized to assist the model in recovering positional and pixel information of the image edges, thereby addressing the noise and fuzzy boundaries characteristic of echocardiograms. Secondly, we propose capturing spatio-temporal correlations by learning optical flow between frames. Finally, leveraging the optical flow learned from two consecutive frames, a temporal consistency module is employed to jointly learn spatio-temporal information by aligning distorted segmentation predictions with actual segmentation predictions at time t. The network was evaluated on the EchoNet-Dynamic dataset [19], the only large-scale echocardiogram dataset available. The contributions of this paper are as follows.

1. We develop a dual-task network comprising a supervised semantic segmentation branch and an unsupervised optical flow learning branch to capture the coherence between consecutive frames.
2. We propose a multi-scale edge-attention U-Net segmentation model, which significantly enhances the model’s ability to segment fuzzy boundaries of the left ventricle.
3. We employ a temporal consistency constraint to jointly train the two branches, enabling the network to learn spatio-temporal features from echocardiograms.
4. The proposed model achieves superior segmentation performance on the EchoNet-Dynamic dataset and demonstrates higher consistency on transition frames compared with existing methods.

## 2. Materials and Methods

### 2.1. Methods

**Figure 1:**
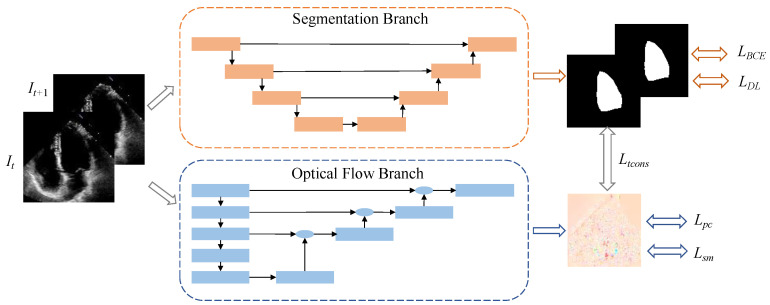
Network structure diagram. The top half represents the segmentation branch, and the bottom half represents the optical flow branch.

In this paper, we introduce a self-attention-based dual-task learning segmentation model, termed MUF-Net, as depicted in Figure 1. The proposed method encompasses two primary tasks: semantic segmentation and optical flow learning. Specifically, the input video frame is processed by the semantic segmentation branch to segment the left ventricular region. To enhance the model’s segmentation capability for the fuzzy edges of the left ventricle, we propose a novel MSEA-U-Net architecture. The optical flow branch is designed to capture the optical flow variations and temporal information between consecutive frame pairs. Ultimately, the two branches are jointly trained to utilize the proposed temporal consistency mechanism.

#### 2.1.1. Overview of Framework Workflow

The overall architecture of our model is a dual-branch network comprising a segmentation branch and an optical flow branch. The EchoNet dataset contains a substantial number of video frames, with only the end-diastolic (ED) and end-systolic (ES) frames labeled in each video. Consequently, during network training, the segmentation branch can utilize only these two labeled frames, while the optical flow branch has access to all frames. This results in an extensive optical flow learning process that spans from the first to the last frame of the video. Even when the video is segmented into shorter clips, such as from one labeled frame to another, optical flow errors can significantly impact segmentation outcomes. This process highlights the strong dependence of segmentation performance on the quality of optical flow learning. Since each video covers four chambers and the optical flow training is unsupervised, the optical flow in echocardiograms focuses on all moving objects, not only the left ventricle. However, with merely two labeled frames, it is challenging to guide the direction of optical flow learning through joint training with the segmentation branch. In this study, during the training phase, we extracted and defined two clips for each video, as its effectiveness has been validated in our previous work [20]: clip 1 consists of the ES frame and its adjacent frames, clip 2 consists of the ED frame and its adjacent frames. They are defined as *c*1: {$I_{ES-1}$, $I_{ES}$, $I_{ES+1}$} and *c*2: {$I_{ED-1}$, $I_{ED}$, $I_{ED+1}$}. As illustrated in Figure 1, all clips are trained in pairs to concurrently learn semantic segmentation and optical flow. During the testing phase, we evaluate all frames of each video and generate the predicted left ventricular mask using solely the segmentation branch. The left ventricular volume of each frame can be approximated by the number of pixels in its predicted mask.

#### 2.1.2. Segmentation Learning

Semantic segmentation of echocardiograms faces two major challenges. First, the EchoNet-Dynamic dataset comprises images with very low resolution (112 × 112). The characteristics of ultrasound imaging result in input feature maps with severe speckle noise and blurred edges [21]. Additionally, 29% and 23% of patients in the dataset have heart failure and coronary artery disease, respectively. These conditions cause ventricular deformation and motion artifacts due to arrhythmias or changes in heart rate during ultrasound scans. Consequently, a robust deep-learning model is required to accurately segment the left ventricle.

**Figure 2:**
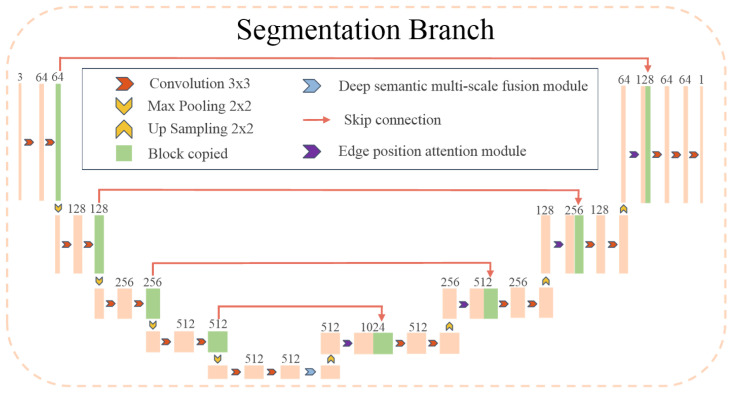
The architecture of the proposed MSEA-U-Net. The blue block indicates the deep semantic multi-scale fusion module (DSMSFM), and the purple block indicates the module-edge position-attention module (EPAM).

To address the aforementioned challenges, the proposed semantic segmentation branch of MSEA-U-Net, referred to as the S-branch, is illustrated in Figure 2. This branch adopts a network architecture similar to the U-Net [22] structure as the baseline. The encoder-decoder design follows a process of first narrowing and then expanding, which enables the model to capture features at multiple levels, ranging from coarse to fine. This multi-level feature capture helps mitigate the interference of noise on segmentation outcomes. The network employs four upsampling operations and utilizes skip connections at the same stage to ensure that the final reconstructed feature map integrates a richer set of features. Features from different scales are fused, resulting in more refined edge information in the segmentation map. In the encoder stage, to enrich the semantic features of the feature maps used for upsampling, we introduce a Deep Semantic Multi-scale Fusion Module (DSMSFM). In the decoder stage, to recover edge information lost during downsampling and feature fusion, we design an Edge Location Attention Module (ELAM).

(1) MSEA-U-Net encoder

**Figure 3:**
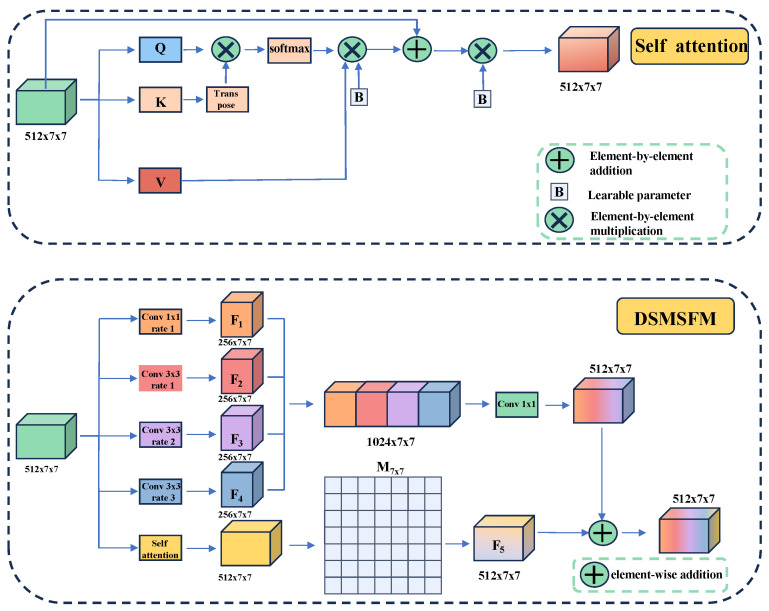
Deep Semantic Multi-scale Fusion Module (DSMSFM). The bottom is the overall structure of DSMSFM, and the top is the details of the self-attention module.

In the semantic segmentation network, the encoder is tasked with continuously performing convolution and downsampling operations on the input to enhance the model’s feature extraction capabilities. However, this process can lead to information loss during convolution or downsampling. Due to the low frame resolution of the EchoNet-Dynamic dataset, the feature map becomes significantly smaller after four downsampling operations by the encoder, while the number of channels increases substantially. At this stage, these channels contain rich semantic information, which we aim to fuse to enhance the model’s representational capacity. To achieve this, we design the DSMSFM, as shown in Figure 3.

Firstly, the input features are further integrated via a 1×1 convolution to obtain the feature map $F_{1}$. Subsequently, three dilated convolutions with different dilation rates (1, 2, and 3) are employed to expand the receptive fields of the convolution kernels. It enables the extraction of local semantic features across multiple scales within the deep semantic context. The corresponding output features are designated as $F_{2}$, $F_{3}$, and $F_{4}$, respectively. Then, a self-attention module, as illustrated atop the DSMSFM in Figure 3, is utilized to capture pixel-level global feature relationships across the feature maps. This module enables the extraction of local features at different scales while preserving the resolution of the feature map and enhancing the flexible expression of element-level global features. The resultant feature map is subsequently passed through a learnable matrix M of size 7×7, initialized to zero, to obtain the feature map $F_{5}$. By assigning a learnable weight to each channel, this process suppresses irrelevant feature channels while assigning greater emphasis to important ones. This mechanism thereby enhances the fusion between the feature maps processed by the self-attention module and those processed by the dilated convolutions. Finally, the four extracted feature maps $F_{i}$ (*i* = 1, 2, 3, 4) are concatenated along the channel dimension, fused using a 1×1 convolution, and then added element-wise to the feature map $F_{5}$ to yield the final output *y*,

(1) $$\begin{array}{c} y=\left(W\left(\mathrm{Cat}[F_{1},F_{2},F_{3},F_{4}]\right)+b\right)+F_{5}, \end{array}$$

where *W* is the weight matrix of the 1×1 convolution kernel, *b* is the bias term, and Cat[] is the concatenation operation along the channel.

For the self-attention mechanism, although the DSMSFM can effectively achieve local multi-scale feature fusion, it does not capture the dependencies between long-range features. To address this limitation, the self-attention module is employed to capture the pixel-level dependencies between the elements of the feature map. It is described as

(2) $$\begin{array}{c} \mathrm{Attention}\left(Q,K,V\right)=\left(\left(\left(\mathrm{softmax}\left(\frac{QK^{T}}{\sqrt{d_{k}}}\right)V\right)*w_{b}+x\right)*\omega_{g}\right), \end{array}$$

where *x* represents the input feature map, *Q* denotes the query vector, *K* represents the key vector, and *V* represents the value vector. The parameters $w_{b}$ and $w_{g}$ are learnable parameters initialized to 1 and are collectively denoted by *B* in Figure 3. By introducing these two learnable parameters, the self-attention module is able to extract features in a more flexible manner.

(2) MSEA-U-Net decoder

The input feature maps of the EchoNet-Dynamic dataset exhibit low resolution (112×112), and the characteristics of ultrasound imaging further contribute to unclear edges in these feature maps. After undergoing multi-layer convolution and four downsampling operations performed by the encoder, the pixel information along the edges tends to be lost. Subsequent information fusion via the DSMSFM can lead to the loss of edge position information, which can negatively impact the recovery of both pixel and positional information of the image edges during the decoding stage.

**Figure 4:**
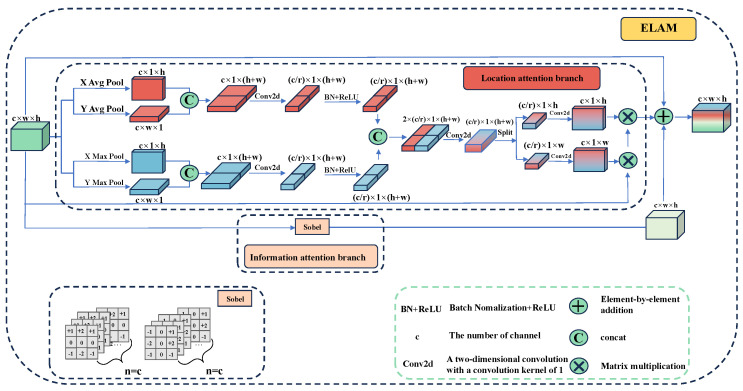
The architecture of Edge Location Attention Module (ELAM). The blue part and the red part are the Location attention branch, and the pink part is the Information attention branch.

To tackle these challenges, this paper introduces the Edge Location Attention Module (ELAM), as depicted in Figure 4. The module consists of two branches. The first branch, known as the location attention branch, focuses on identifying and enhancing edge features within the low-resolution feature map. The second branch, referred to as the information-attention branch, employs traditional operators to detect the edge-pixel information of the feature map. Ultimately, the two feature maps, which contain edge position information and pixel information, respectively, are combined element-wise to generate a feature map that integrates both edge position and pixel information for subsequent upsampling.

a. Location Attention Branch

Attention mechanisms are usually employed to direct the model’s focus on specific content and locations within the data. For instance, the Squeeze-and-Excitation (SE) attention proposed by SENet [23] computes the channel attention mechanism through 2D global pooling. However, the SE module primarily focuses on the information encoding between channels, neglecting the significance of spatial location information. For the echocardiography segmentation, if the model can emphasize the positional information of image edges during the segmentation process, it can enhance the segmentation performance of the model. As illustrated in Figure 4, to mitigate the loss of positional information caused by 2D global average pooling, we employ two one-dimensional global average pooling operations and two max-pooling operations. They are applied along the vertical and horizontal axes, respectively, to aggregate the input features into two distinct direction-aware feature maps. Specifically, for the input *x*, pooling kernels of size (*H*, 1) and (1, *W*) are first applied to encode each channel along the horizontal and vertical coordinate directions, respectively, as shown in Equations (3)–(6).

(3) $$\begin{array}{c} a_{c}^{h}\left(h\right)=\frac{1}{H}\sum_{0\leq i<H}x_{c}\left(h,i\right) \end{array}$$

(4) $$\begin{array}{c} a_{c}^{w}\left(w\right)=\frac{1}{W}\sum_{0\leq j<W}x_{c}\left(j,w\right) \end{array}$$

(5) $$\begin{array}{c} m_{c}^{h}\left(h\right)=\max_{0\leq i<H}x_{c}\left(h,i\right) \end{array}$$

(6) $$\begin{array}{c} m_{c}^{w}\left(w\right)=\max_{0\leq i<W}x_{c}\left(j,w\right) \end{array}$$

Let $a_{c}^{h}\left(h\right)$ denote the output after the average pooling of the *c-th* channel along the height dimension *h*, $a_{c}^{w}\left(w\right)$ denote the output after the average pooling of the *c-th* channel along the width dimension *w*, $m_{c}^{h}\left(h\right)$ denote the output after max-pooling of the *c-th* channel along the height dimension *h*, and $m_{c}^{w}\left(w\right)$ denote the output after max-pooling of the *c-th* channel along the width dimension *w*. Subsequently, each pair of obtained feature maps is concatenated along the second dimension and then transformed using a shared 1×1 convolution to facilitate information sharing. They are defined as

(7) $$\begin{array}{c} f_{a}=\delta \left({\mathrm{Conv}}_{1\times 1}\left(\mathrm{Cat}\left[a^{h},a^{w}\right]\right)\right), \end{array}$$

(8) $$\begin{array}{c} f_{m}=\delta \left({\mathrm{Conv}}_{1\times 1}\left(\mathrm{Cat}\left[m^{h},m^{w}\right]\right)\right), \end{array}$$

where the outputs $f_{a}\in R^{C/r\times H}$ and $f_{m}\in R^{C/r\times H}$ are intermediate feature maps with spatial information in horizontal and vertical directions, respectively. *r* is the downsampling ratio, we set *r* to 16 in this paper accordingly [24]. ${\mathrm{Conv}}_{1\times 1}$ is the 1×1 convolution, $a^{h}\in R^{1\times h\times c}$, $m^{h}\in R^{1\times h\times c}$ and $a^{w}\in R^{w\times 1\times c}$, $m^{w}\in R^{w\times 1\times c}$ are the feature maps after vertical and horizontal pooling, respectively, and δ is BatchNorm and ReLU.

Then, the two output feature maps are concatenated along the channel. In addition, the 1×1 convolution is used for feature fusion to obtain the feature figure $f\in R^{\left(C/r\times H)\times 1\times (h+w\right)}$, and the feature fusion after different pooling processing is realized. It can be presented as

(9) $$f=Conv_{1\times 1}\left(Cat\left[f^{a},f^{m}\right]\right).$$

Subsequently, *f* is split into two separate tensors $f^{h}\in R^{(C/r\times H)\times h\times 1}$ and $f^{w}\in R^{(C/r\times W)\times 1\times w}$ along the spatial dimension, and then $f^{h}\in R^{(C/r\times H)\times h\times 1}$ is transformed to ensure that $f^{h}$ is the attention feature map in the *h* direction. We then use two 1×1 convolutions to map the feature map to the same number of channels as the input feature. This process is defined as

(10) $$f^{h},f^{w}=\mathrm{split}\left(f,\left[H,W\right],\dim=2\right),$$

(11) $$f^{h}=\mathrm{permute}\left(f^{h},\left[0,1,3,2\right]\right),$$

(12) $$g^{h}=\sigma \left({\mathrm{Conv}}_{1\times 1h}\left(f^{h}\right)\right),$$

(13) $$g^{w}=\sigma \left({\mathrm{Conv}}_{1\times 1w}\left(f^{w}\right)\right),$$

where $g^{h}\in R^{c\times h\times 1}$ and $g^{w}\in R^{c\times 1\times w}$ of the outputs are the attention weights of the input feature map in the height direction and width direction. $Conv_{11w}$ and $Conv_{11h}$ are the 1×1 convolution, and σ is the sigmoid activation function. Finally, the final feature map with attention weights in width and height direction is obtained by multiplicative weighting calculation on the original feature map, which is calculated as

(14) $$y_{c}\left(i,j\right)=x_{c}\left(i,j\right)\times g_{c}^{h}\left(i\right)\times g_{c}^{w}\left(j\right).$$

b. Information Attention Branch

The edge of an image is typically defined as the point where there is a significant transition in pixel values, corresponding to the maximum of the first derivative. To detect such edges, we compute the first derivative of the image as

(15) $$f^{\prime }\left(x\right)=f\left(x\right)-f\left(x-1\right).$$

A larger $f^{\prime }\left(x\right)$ indicates more significant pixel changes in the x-direction, suggesting stronger edge signals. The Sobel operator is a discrete differential operator designed to compute the approximate gradient of image grayscale levels. A higher gradient value implies a higher likelihood of an edge.

The Sobel operator [25] consists of two specific 3×3 convolution kernels. By convolving the image with these kernels, gradient images in both the *X* and *Y* directions are obtained, as calculated by Equations (17) and (18), respectively. The horizontal and vertical grayscale values of each pixel in the image are then combined using Equation (18) to calculate the resultant grayscale value at that point.

(16) $$G_{x}=\left[\begin{array}{ccc} -1 & 0 & +1\\ -2 & 0 & +2\\ -1 & 0 & 1 \end{array}\right]*A$$

(17) $$G_{y}=\left[\begin{array}{ccc} +1 & +2 & +1\\ 0 & 0 & 0\\ -1 & -2 & -1 \end{array}\right]*A$$

(18) $$G_{x,y}=\sqrt{G_{x}^{2}+G_{y}^{2}}$$

Let $G_{x}$ represent the gradient image in the *x*-direction, $G_{y}$ represent the gradient image in the *y*-direction, and A denote the feature map. In this paper, the Sobel operator is applied to each channel of the input feature map. Through the Sobel operator and convolution operation, the pixel information of the edge of the feature map is obtained. This process helps the feature map focus more on the edge information when it is upsampled back to the original image size. Finally, a residual block is introduced to enhance the robustness of the model, which is defined as

(19) $$y=G_{x,y}+y_{c}\left(i,j\right)+x,$$

where *y* is the output of the Edge Location Attention Module, *G* is the result of the information attention branch, $y_{c}$(*i*,*j*) is the output of the location attention branch, and *x* is the input feature map.

The segmentation branch uses two common semantic segmentation loss functions: binary cross-entropy (*BCE*) loss and Dice loss function (*DL*), which are defined as

(20) $$L_{BCE}=-y\log\hat{y}-\left(1-y\right)\log\left(1-\hat{y}\right),$$

where *y* and $\hat{y}$ represent semantic region labels and prediction results, respectively.

(21) $$L_{Dice}=1-\frac{2|Y\cap Y^{\prime }|}{\left|Y|+\right|Y^{\prime }|}$$

We set the predicted segmentation result as *Y* and its corresponding label as $Y^{\prime }$; The numerator is twice the overlap area of the two sets *Y* and $Y^{\prime }$, and the denominator is the sum of the elements in the two sets. It is presented as

(22) $$L_{S}=L_{BCE}+L_{Dice}.$$

#### 2.1.3. Optical Flow Learning

For the optical flow branch, we utilize a specialized network to capture the temporal information between two adjacent frames through optical flow estimation. Compared to region-based networks, a pixel-wise algorithm is more suitable for identifying pixel-level motion between consecutive frames. Specifically, most brightness changes occur at the edges of the heart chambers, which also aids in distinguishing these edges from the background.

**Figure 5:**
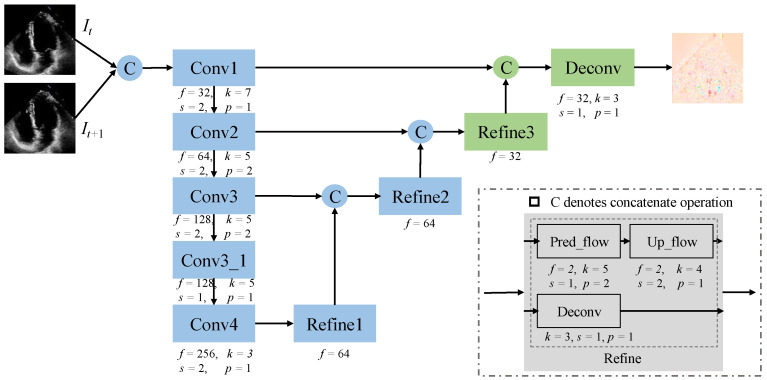
The architecture of the modified FlowNet (mFlowNet). The blue rectangles represent the original FlowNet blocks, while the green rectangles represent the modified parts by ours.

In this section, we design a modified FlowNet based on FlowNetSimple [26]. Figure 5 illustrates the architecture of the improved FlowNet, denoted as mFlowNet. The blue component comes from the original FlowNetSimple, we customize it by importing some layers. The green part represents our modification, where we add more upsampling layers to ensure that the output has the same size as the input. The reason for this is that we want to use deconvolution to learn the upsampling process instead of using interpolation during the warping computation. The hyperparameters corresponding to each operation are shown in Figure 5 below, where f represents the number of features, k represents the kernel size of the convolution, s represents the step size, and p represents the padding size. The number of features deconvolution in the refinement operation is specified below the refinement block. “Uplink traffic” represents an uplink sampling operation to predict traffic. In mFlowNet, we also adopt an encoder and decoder structure to learn the optical flow between every two frames. It contains five normal convolution and downsampling blocks in the encoder. For the decoder, we introduce two additional upsampling layers and a feature fusion layer to ensure that the output size matches the input size.

We denote the optical flow branch as $O_{p}$(*x*), where p represents its corresponding parameters, and simply refer to it as the *O*-branch. The input to the *O*-branch is pairwise and identical to that of the *S*-branch. The output of mFlowNet is the optical flow between the two input frames, denoted as $M_{t\to t+1}$. mFlowNet is trained in an unsupervised manner, with its updates relying on the fundamental features of optical flow, photometric consistency, and motion smoothness.

Photometric consistency loss [27,28] is to constrain a frame and the warped image from its adjacent frame, which is defined as

(23) $$L_{pc}=\alpha \frac{1-SSIM(I-I_{w})}{2}+\left(1-\alpha \right){∥I-I_{w}∥}_{1},$$

where $I_{w}$ is the warped image, *SSIM* is the structural similarity index and α is set to 0.85 accordingly [28]. The purpose of motion smoothness is intended to eliminate erroneous predictions while preserving crisp details, which is defined as

(24) $$L_{sm}=\sum_{x,y}\left|\nabla M\left(x,y\right)\right|\cdot \left(e^{-|\nabla I(x,y)|}\right),$$

where ∇ is the vector differential operator, |·| denotes element-wise absolute value. The total loss function for the O-branch is presented as

(25) $$L_{O}=\lambda_{1}L_{pc}+\lambda_{2}L_{sm},$$

where $\lambda_{1}$ and $\lambda_{2}$ are the corresponding weights of two losses, respectively.

#### 2.1.4. Cooperation Mechanism and Joint Learning

For the two branches described above, the S-branch is designed to learn spatial semantic features, while the O-branch is tasked with capturing temporal features between frames. To further enhance segmentation performance, we employ a temporal consistency constraint to integrate the learned features from both branches. We adopt the temporal consistency model introduced in [29]. In their work, the temporal consistency constraint was defined as a function of the encoder output features at time t and the distorted features at time *t* + 1. However, in this paper, we redefine the temporal consistency constraint as a function of the segmentation output at time t and the distorted output at time *t* + 1, leveraging the optical flow learned from the O-branch. The rationale for this approach is rooted in the nature of ultrasound imaging, where the edges between the left ventricle and the background are often blurred. Consequently, a temporal consistency module that processes only the segmentation output can effectively filter out background noise and irrelevant information in regions outside the left ventricle. Since the segmentation output is binary—with background pixel values set to zero—only the segmented left ventricular region is utilized in the optical flow warping calculations.

Given a pair of input frames $I_{t}$ and $I_{t+1}$, we obtained their semantic segmentation results from branch *S*, $Y_{t}$ and $Y_{t+1}$, respectively, and obtained their predicted optical flow from branch *O*, $M_{t}$ and $M_{t+1}$. Then we warped $Y_{t}$ to $Y_{t+1}$ by optical flow $M_{t\to t+1}$, which are calculated as

(26) $$Y_{t}^{\prime }=\mathrm{Warp}\left(Y_{t+1},M_{t\to t+1}\right),$$

where we also used differentiable bilinear interpolation for warping. The temporal consistency loss is defined as

(27) $$L_{tcons}=\sum_{x,y}∥Y^{\prime xy}-Y^{xy}∥.$$

By employing optical flow and warping, we effectively integrate temporal features into the spatial domain. This allows us to leverage the *O*-branch to extract features from unlabeled frames and subsequently enhance the semantic segmentation results through warping. The two branches train in an end-to-end manner, collaboratively achieving video segmentation and thereby improving the overall performance of the model. The total loss function of the proposed model is

(28) $$L=L_{S}+L_{O}+\lambda_{3}L_{tcons}=L_{BCE}+L_{Dice}+\lambda_{1}L_{pc}+\lambda_{2}L_{sm}+\lambda_{3}L_{tcons},$$

where the weights of $L_{S}$ and $L_{O}$ are set to 1, the weights of $L_{tcons}$ is $\lambda_{3}$.

### 2.2. Materials

The EchoNet-Dynamic dataset, utilized in this study, is a large-scale, publicly available echocardiogram video dataset designed for cardiac function assessment. It comprises 10,030 echocardiographic videos, each independently recorded from 10,030 individuals. To protect patient privacy, all videos are saved in AVI format. These 2D grayscale videos are captured from the apical 4-chamber view. The number of frames per video ranges from 28 to 1002, with an average frame rate exceeding 176 frames per second. For each video, the dataset provides the video length, the temporal locations of the ES and ED frames, corresponding masks and volumes, and the computed EF. All frames in the dataset have a resolution of 112 × 112 pixels. Annotations were provided by experienced experts.

The experiments were conducted using the PyTorch library, version 2.0.1. Training and testing were performed on a machine equipped with an Intel Core i9-13900K CPU, 62 GB of memory, and a GeForce RTX 4090 Ti 24GB GPU, running the Ubuntu 22.04 operating system.

The dataset was divided into training, validation, and test sets in the proportions of 75%, 12.5%, and 12.5%, respectively, consistent with the original EchoNet-Dynamic dataset [30]. For a fair comparison with other models, we also evaluated our method using an 80% training and 20% testing split. During training, as previously described, we used video clips to train the proposed model, generating four input pairs per video. In the testing phase, we evaluated all frames in each video. Due to memory constraints, the model was trained for 100 epochs with a batch size of one. Model weights were updated using the Adam optimizer with an initial learning rate of $1.6\times 10^{-5}$. For the loss function, we experimentally set the weights $\lambda_{1}$, $\lambda_{2}$, and $\lambda_{3}$ to 5, 0.2, and 0.4, respectively. In this work, we evaluated the segmentation performance of the proposed model using the Dice coefficient score and Hausdorff distance (HD). The Dice score, related to the Dice loss, is defined as follows:

(29) $$\mathrm{Dice}\left(Y,G\right)=1-L_{Dice}.$$

HD is used to estimate the maximum distance between the predicted value and the ground truth, which is defined as

(30) H(Y,G)=max(h(Y,G),h(G,Y)).

Taking the direct Hausdorff distance as an example, it is expressed as

(31) $$h\left(Y,G\right)=\max_{y\in Y}\left(\max_{g\in G}\left(d\left(y,g\right)\right)\right),$$

where d(y,g) represents the Euclidean distance between *y* and *g*.

## 3. Experiment Results

We evaluate the performance of the proposed model from two aspects. First, through ablation experiments, we verify the effectiveness of incorporating temporal features into the spatial feature extraction network for left ventricular segmentation. We then validate the efficacy of the proposed MSEA-U-Net model. Second, we conduct a quantitative analysis to demonstrate the superiority of our proposed method by comparing it with existing networks on the EchoNet-Dynamic dataset. Furthermore, we perform a qualitative analysis of the proposed model through visual experiment comparisons.

### 3.1. Ablation Experiment

#### 3.1.1. Evaluation of Introducing Optical Flow Branch

We evaluated the effectiveness of introducing the optical flow learning auxiliary task by comparing it to a spatial semantic network based on the U-Net architecture. The comparison results are presented in Table 1. It reveals that the implementation of the optical flow branch results in a 3.75% improvement in the segmentation performance of the network compared to the U-Net. Additionally, compared with FlowNetSimple, our optical flow branch model not only ensures consistency in output size between the optical flow and the input but also extracts more detailed optical flow field information, resulting in a 0.14% increase in the Dice score.

The results demonstrate that the simultaneous extraction of spatial and temporal features is significantly more advantageous for echocardiographic video segmentation than the extraction of spatial features alone. The temporal features capture the information between adjacent frames, enabling the model to not only perform semantic segmentation of the left ventricle but also learn the spatio-temporal changes between these frames. Since the optical flow branch is trained in an unsupervised manner, it can learn the attributes of unlabeled frames, thereby extracting a substantial amount of characteristic information from these frames. This learned optical flow field transformation between two adjacent frames can then guide the segmentation of transition frames, ensuring the accuracy and consistency of segmentation across each frame of the video.

#### 3.1.2. Evaluation of the Proposed MSEA-U-Net Semantic Segmentation Model

By comparing the U-Net + mFlowNet and MSEA-U-Net + mFlowNet models in Table 1, it is evident that the MSEA-U-Net semantic segmentation model proposed in this paper achieves higher performance than U-Net. The introduction of the DSMSFM in the encoder stage and the EPAM in the decoding stage gives the model the ability to identify the more accurate boundary of the left ventricle. The experimental results demonstrate that the proposed MSEA-U-Net can effectively achieve a multi-scale fusion of deep semantic features, thereby enhancing the model’s representational capacity in the decoding stage. The EPAM is proposed in the decoding stage. In addition, it also can accurately locate edge positions and recover pixel information during the decoding process.

### 3.2. Comparison with Existing Methods

We quantitatively and qualitatively evaluate the segmentation performance of our proposed model with existing 2D frame segmentation and video-based segmentation methods on the EchoNet-Dynamic dataset.

1. For 2D ES and ED frames segmentation methods, we compare with the primary algorithm proposed by Ouyang et al., the **EchoNet-Dynamic** method [30], and three recent models which combined transformer module or attention mechanism: TransBridge [8] (which offers **TransBridge-B** and **TransBridge-L** variants), **PLANet** [31], and **Bi-DCNet** [12].
2. For the echocardiographic video segmentation algorithms, we compare with the **Joint-Net** [32] and **BSSF-NET** [18], and **JASO-Net** [20]. **This article serves as an extension of JASO-Net.** BSSF-NET employs a two-way spatio-temporal semantic fusion technique instead of optical flow.

#### 3.2.1. Quantitative Analysis

To quantitatively demonstrate the advancement of our model, we conducted a comparative evaluation with the above existing methods, as shown in Table 2. Among these methods, the comparison results for EchoNet-Dynamic, TransBridge-B, TransBridge-L, and Bi-DCNet were extracted from their respective publications. The training, testing, and validation sets they used are assigned by the EchoNet-Dynamic dataset with a ratio of 75:12.5:12.5. Here, we call it ratio-1 for convenience. Notably, the three sets of the first ratio are fixed, so we did not implement cross-validation but applied the validation set. Consequently, we did not demonstrate the standard deviation (STD) in Table 2. In contrast, the results for PLANet, Joint-Net, and BSSF-NET were obtained from the BSSF-NET paper. The training and test sets they used are randomly selected from the EchoNet-Dynamic dataset with a ratio of 80:20, denoted as ratio-2. They are evaluated using 5-fold cross-validation without separate validation sets. Since we have evaluated our proposed JASO-Net using both ratios, we focus on presenting the results for ratio-1 in this work.

In Table 2, our previously developed JSAO-Net exhibited superior segmentation performance compared to existing methods. Moreover, the newly proposed MUF-Net architecture in this study demonstrates additional enhancements in the Dice score over its predecessor and other comparative models. These results indicate that our proposed MSEA-U-Net segmentation branch excels at identifying the fuzzy edges of the left ventricle. Furthermore, compared with the 2D image segmentation methods and strategies, the joint learning of semantic features and optical flow in our model more effectively leverages spatio-temporal information. The optical flow field captures the changes in optical flow between different frames, and the results of optical flow encompass the spatio-temporal transformation between these frames. Subsequently, the results of optical flow and semantic segmentation are combined through temporal consistency, which guides the semantic segmentation task to learn more effective features and achieve better segmentation outcomes. It also can be seen that the HD index in this work is the third among the compared models, which means there are outliers in our segmentation results. However, the segmentation study in this paper is for the LVEF assessment, which is calculated based on ESV and EDV estimated by the areas of ES and ED. The impact of outliers on LVEF calculation is limited.

We evaluated the computational complexity of the model from three aspects: number of parameters, inference speed, and floating-point operations per second (Flops), and the results are shown in Table 3. It reveals that although the introduction of the MSEA-U-Net encoder increases the parameter number of the model and introduces additional complexity to the model, the inference speed is improved. This indicates that the design MSEA-U-Net encoder allows the model to process information more efficiently, leading to faster inference times despite the increased complexity.

#### 3.2.2. Qualitative Analysis

**Figure 6:**
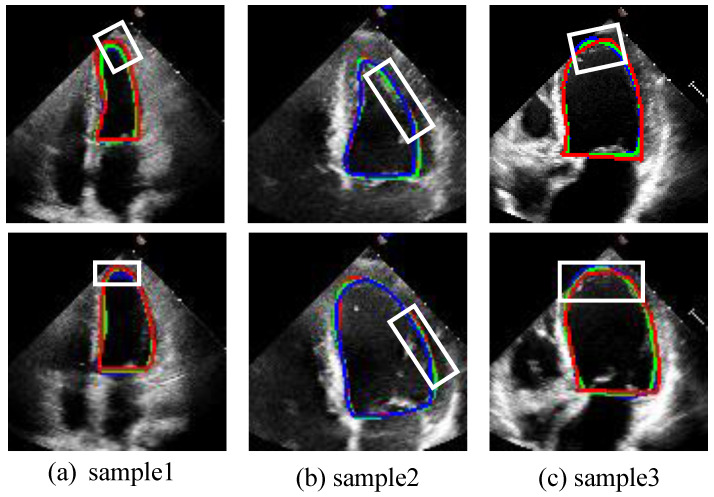
The segmentation results of the ES frame and ED frame. From (**a**–**c**), each column is a sample of the ES and ED frames in the video. The green line is the result of this work. The blue line is the result of the contrast algorithm (JSAO-Net), and the red line is the label. The white box presents the main difference between the segmentation results of the two algorithms.

To save space, this paper visually compares our proposed model with the JSAO-Net model, which exhibited the best performance among the comparison algorithms, as depicted in Figure 6. For clarity, we present the segmentation contour results, where the red circle denotes the ground truth label, the blue circle represents the segmentation result of the comparison algorithm, and the green circle indicates the segmentation result of our proposed model. As evident from the white box in Figure 6, our proposed model achieves more accurate edge segmentation compared to JSAO-Net [20], demonstrating superior precision in delineating the left ventricular boundary. The segmentation branch in JASO-Net merely employs a U-Net-like structure without proposing specific strategies to address the fuzzy edges of the left ventricle. In contrast, the DSMSFM and ELAM introduced in our model significantly enhance semantic fusion and edge information attention, outperforming JSAO-Net in these aspects.

**Figure 7:**
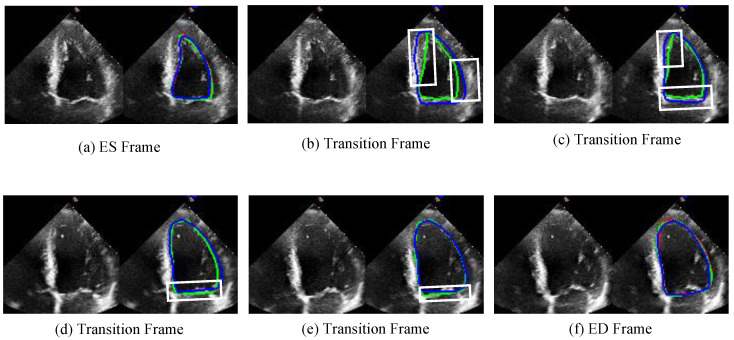
The comparison results of unlabeled transition frames are shown in the figure. (**a**,**f**) are ES and ED frames, respectively, and (**b**–**e**) are four transition frames between them. The left side of each image shows the original image and the right side shows the corresponding comparison visualization. The green line is the result of this work. The blue line is the result of the JSAO-Net, and the red line is the label. The white box presents the main difference between the segmentation results of the two algorithms.

Figure 7 illustrates the segmentation capability of our proposed algorithm on transition frames. The figure displays the segmentation results of two video clips, with frames ordered from left to right and then from the first row to the second row. The first frame corresponds to the ES frame, and the last frame corresponds to the ED frame. Additionally, four transition frames are included for each video clip. For each frame, the left side shows the original frame, while the right side presents the segmentation contour result. As shown in Figure 7, Our model not only demonstrates superior performance on ES and ED frames but also achieves remarkable segmentation results on transition frames. The white box highlights that the JSAO-Net model tends to overshoot the segmentation boundary, whereas our model exhibits more stable and accurate performance. The segmentation of transition frames relies on both semantic segmentation and optical flow learning. In this study, the semantic segmentation branch extracts multi-scale edge features of the left ventricle to enhance robustness against the fuzzy edges characteristic of ultrasound images. Subsequently, spatio-temporal information transfer between adjacent frames is achieved through the optical flow field changes learned by the optical flow branch. This integrated process not only improves the segmentation accuracy of key frames but also enhances the precision and consistency of transition frame segmentation.

In summary, this algorithm achieves joint spatio-temporal learning of the left ventricle in echocardiography by employing a dual-task network that combines semantic segmentation and optical flow learning. Utilizing the proposed MSEA-U-Net architecture, our approach enhances the model’s ability to recognize the fuzzy edges of the left ventricle, leading to improved performance in video segmentation tasks. When compared to existing algorithms, our method not only demonstrates superior segmentation accuracy for ES and ED frames but also exhibits more stable and precise segmentation capabilities across intermediate frames.

## 4. Discussion

In this study, we introduce a novel method for automatic left ventricular segmentation based on a self-attention mechanism with dual-task learning. The first task involves learning the optical flow fields between consecutive frames using an optical flow branch, while the second task focuses on supervised semantic segmentation. To enhance the model’s ability to segment fuzzy boundaries of the left ventricle, we propose a multi-scale edge-attention U-Net segmentation model. The two tasks are jointly trained using a temporal consistency constraint. We validated our approach on the EchoNet-Dynamic dataset, the largest available echocardiographic dataset. The experimental results demonstrate that our method achieves superior segmentation performance for left ventricular ED and ES frames, as well as transition frames. This capability is crucial for accurately estimating left ventricular volume in clinical settings and provides an important tool for the calculation of ejection fraction.

Existing echocardiographic segmentation algorithms primarily focus on single-frame segmentation of ED and ES frames, neglecting the temporal correlations between consecutive frames. However, manually labeling each frame is extremely costly. Therefore, in our study, we propose using optical flow to capture the changes in the optical flow field between frames in an unsupervised manner. This approach allows us to obtain the temporal connections between frames without increasing labeling costs, and the segmentation branch is guided through joint training. To achieve better segmentation results, current semantic segmentation networks for echocardiography have introduced various pyramid fusion modules and attention mechanisms to enhance feature extraction and fusion capabilities [33,34,35]. However, these methods do not specifically address the fuzzy edges characteristic of echocardiographic images. In the encoder stage, we propose a DSMSFM to achieve a multi-scale fusion of rich semantic features. In the decoder stage, we introduce an EPAM to better recover edge information through the combined efforts of edge position attention and edge-pixel information-attention modules.

Our approach excels by leveraging frame-to-frame spatio-temporal characteristics and employing a targeted strategy for the fuzzy edges of the left ventricle. However, our approach has certain limitations. If a patient has heart disease, it may cause ventricular deformation or arrhythmias, which can affect the learning performance of the optical flow branch. Additionally, noise in echocardiograms affects not only the optical flow branch but also the segmentation branch.

Automatic left ventricle segmentation is an important step for LVEF evaluation, as it enables beat-by-beat estimation of ESV and EDV. Through the segmentation, we can calculate LVEF using the mean value of all beats, which follows the clinical convention, particularly in conditions like heart failure and cardiomyopathy. Improving the accuracy of left ventricular segmentation enhances the reliability of ESV and EDV measurements, leading to more precise LVEF calculations. Given that LVEF is a key indicator for assessing cardiac function and guiding clinical treatment decisions, improving the accuracy of left ventricular segmentation is a crucial step in enhancing the precision of LVEF assessment and holds significant clinical importance. Based on the segmentation result, we obtained the LVEF through beat-by-beat measurement, where LVEF is a crucial index to assess cardiac function in clinical practice. To show the clinical relevance of improved segmentation accuracy, as shown in Table 4, we use two indexes, *p*-value and Cohen’s d. In clinical research, *p*-values determine whether results are statistically significant, meaning the observed differences are unlikely due to random factors, with values below 0.05 typically considered significant. Cohen’s d measures the magnitude of the effect by standardizing the difference in means between two groups, helping to assess the practical significance of the results. According to Cohen’s guidelines, effect sizes of 0.2, 0.5, and 0.8 represent small, medium, and large effects, respectively. Hence, *p*-values and Cohen’s d provide a more comprehensive evaluation of the clinical relevance of the improvement in segmentation performance and corresponding enhancement in LVEF. It can be seen that the *p*-values and Cohen’s d values are very similar among the three models, where *p*-values are smaller than 0.05, and Cohen’s d values are larger than 0.8. It means that automatic segmentation algorithms can achieve more reliable LVEF estimation, and has a positive impact on diagnosis and assessment in clinical practice.

In future studies, we can explore the characteristics of ventricular deformation and constrain the learning of optical flow by introducing prior knowledge or incorporating a shape loss function during the loss calculation stage. To address the impact of noise, we can investigate more advanced feature fusion methods, such as employing additional self-attention mechanisms or larger receptive field attention mechanisms. Since segmentation results at the pixel level are influenced by multiple factors, these approaches can help mitigate the impact of noise on segmentation outcomes.

Although our method has achieved promising results in left ventricular segmentation of echocardiograms, future research will continue to explore more advanced left ventricular segmentation methods, with a particular focus on fully utilizing the periodicity of the heartbeat and effectively addressing the noise inherent in echocardiographic images.

## 5. Conclusions

In this paper, we present a novel video segmentation network for echocardiography, MUF-Net, developed based on the EchoNet-Dynamic dataset. The network has a dual-task architecture that includes semantic segmentation and optical flow learning. For semantic segmentation, we propose a multi-scale edge-attention U-Net to enhance the model’s ability to segment the fuzzy edges of the left ventricle. The optical flow learning task captures the changes in the optical flow field between frames, thereby enabling accurate segmentation of adjacent frames. The two branches collaborate to integrate spatial and temporal information from the video using a temporal consistency module, thereby improving the performance of left ventricular segmentation. Experimental results demonstrate that our model outperforms both 2D ES and ED frame segmentation methods and existing echocardiographic video segmentation methods, achieving a Dice score of 92.71%. The performance of our algorithm is more stable and reliable compared to other algorithms. In future work, we will explore more advanced temporal feature extraction strategies and fusion mechanisms to further enhance the model’s segmentation performance.

## Figures and Tables

**Table 1:** Ablation experiment. Bold font indicates the best result.

Structure	Dice Score (%)
U-Net	88.76
U-Net + FlowNetSimple	92.50
U-Net + mFlowNet	92.64
U-net + mFlowNet + MECAM (this work)	**92.71**

**Table 2:** Comparison result with existing methods. Bold font indicates the best result.

Methods	Year	Train/Val/Test: 75/12.5/12.5		Train/Val/Test: 80/-/20	
		Dice Score (%)	HD (mm)	Dice Score (%) (Mean ± STD)	HD (mm) (Mean ± STD)
EchoNet-Dynamic	2020	91.97	2.32	93.79 ± 0.22	2.27 ± 0.47
Joint-net	2020	-	-	90.91 ± 0.36	3.85 ± 0.92
TransBridge-B	2021	91.39	4.41	-	-
TransBridge-L	2021	91.64	4.19	-	-
PLANet	2021	-	-	91.92 ± 0.34	3.42 ± 0.67
BSSF-Net	2022	-	-	92.87 ± 0.16	2.93 ± 0.72
Bi-DCNet	2023	92.25	-	-	-
JSAO-Net	2024	92.64	**2.23**	96.99 ± 0.12	1.76 ± 0.47
Ours	2025	**92.71**	3.85	**-**	**-**

**Table 3:** Computational complexity comparison with the existing methods.

Method	Parameter (M)	Speed (*ms/f*)	Flops (G)
Echonet-Dynamic	39.60	14	7.85
Joint-net	117.27	62	108.32
TransBridge-B	**3.49**	-	-
TransBridge-L	11.30	-	-
PLANet	20.75	34	74.95
BSSF-Net	74.79	32	56.36
JSAO-Net	17.27	9	**7.69**
Ours	24.70	**8**	68.65

**Table 4:** Clinical relevance of improved segmentation accuracy.

Method	Dice Score (%)	*p*-Value	Cohen’s d
Echonet-Dynamic	91.97	$2.62\times 10^{-12}$	1.073
JSAO-Net	92.64	$1.13\times 10^{-13}$	1.041
Ours	92.71	$9.82\times 10^{-11}$	1.044

## Data Availability

Data are contained within the article.
